# Neural correlates of transfer of learning in motor coordination tasks: role of inhibitory and excitatory neurometabolites

**DOI:** 10.1038/s41598-024-53901-8

**Published:** 2024-02-08

**Authors:** Amirhossein Rasooli, Sima Chalavi, Hong Li, Caroline Seer, Hamed Zivari Adab, Dante Mantini, Stefan Sunaert, Mark Mikkelsen, Richard A. E. Edden, Stephan P. Swinnen

**Affiliations:** 1 Movement Control and Neuroplasticity Research Group, Department of Movement Sciences, Group Biomedical Sciences, KU Leuven, Leuven, Belgium; 2 Leuven Brain Institute (LBI), KU Leuven, Leuven, Belgium; 3 Department of Imaging and Pathology, Group Biomedical Sciences, KU Leuven, Leuven, Belgium; 4 Department of Radiology, Weill Cornell Medicine, New York, NY USA; 5 Russell H. Morgan Department of Radiology and Radiological Science, The Johns Hopkins University School of Medicine, Baltimore, MD USA; 6 F. M. Kirby Research Center for Functional Brain Imaging, Kennedy Krieger Institute, Baltimore, MD USA; 7 Motor Control Laboratory, Movement Control and Neuroplasticity Research Group, KU Leuven, Tervuurse Vest 101, Building De Nayer, Room 02.11, 3001 Leuven, Belgium

**Keywords:** Transfer of learning, Bimanual coordination, Magnetic resonance spectroscopy, GABA, Glx, Motor control, Learning and memory

## Abstract

We aimed to investigate transfer of learning, whereby previously acquired skills impact new task learning. While it has been debated whether such transfer may yield positive, negative, or no effects on performance, very little is known about the underlying neural mechanisms, especially concerning the role of inhibitory (GABA) and excitatory (Glu) (measured as Glu + glutamine (Glx)) neurometabolites, as measured by magnetic resonance spectroscopy (MRS). Participants practiced a bimanual coordination task across four days. The Experimental group trained a task variant with the right hand moving faster than the left (Task A) for three days and then switched to the opposite variant (Task B) on Day4. The control group trained Task B across four days. MRS data were collected before, during, and after task performance on Day4 in the somatosensory (S1) and visual (MT/V5) cortex. Results showed that both groups improved performance consistently across three days. On Day4, the Experimental group experienced performance decline due to negative task transfer while the control group continuously improved. GABA and Glx concentrations obtained during task performance showed no significant group-level changes. However, individual Glx levels during task performance correlated with better (less negative) transfer performance. These findings provide a first window into the neurochemical mechanisms underlying task transfer.

## Introduction

Transfer of learning refers to the application of knowledge and skills learned in one situation to solve a similar or related problem in a new situation^1,2^. This broad definition can be applied to various domains of learning, including motor skill acquisition. Acquiring a new motor skill can either start from a point where the individual does not have any prior knowledge or experience in the task at hand, or it can be the generalization or modification of previously acquired skills with alterations in the movement specifications^3^. The nature and degree of transfer depend on the relationship between the already-acquired skills and the to-be-learned ones^4^. For instance, learning how to drive a car requires substantial practice to become proficient. Suppose that an individual learns how to drive in the European Community or the US, where steering wheels are placed on the left side. When this individual subsequently has to drive a car in the UK, i.e., with a steering wheel placed on the right side, transfer of driving skills is expected, even though disruptions in performance may occur due to the unfamiliar positioning of the steering wheel and/or altered positioning of the controls.

Here, we focus specifically on the inter-manual transfer of learning due to its vast applications in daily life to improve our understanding of the behavioral and neural mechanisms underlying transfer.

Inter-manual skill transfer can occur in both unimanual and bimanual task conditions. In unimanual tasks, a trained skill can be transferred from one limb or limb segment to the other. In bimanual tasks, the elements of the task may be similar or closely related, but the ultimate task specifications for each hand may be different (see the aforementioned driving example)^5^. In both uni- and bimanual tasks, positive and/or negative transfer has been observed. *Positive transfer* denotes a scenario in which a previously acquired skill facilitates the performance of the new task. Several studies have demonstrated the positive effect of training with one hand on the performance of the other hand in sequence learning tasks^6,7^. Furthermore, Van Mier and Petersen^8^ investigated the inter-manual transfer of learning in a maze-solving task and found evidence for positive transfer of learning. The authors suggested that this observation signifies the encoding of skills at an abstract level, independent of the limb that is used to carry out the required action^8^. However, negative transfer also occurs whereby pre-existing knowledge or skills may not necessarily facilitate the acquisition of new skills^9–11^. For example, Vangheluwe, Suy^9^ and coworkers required participants to first practice a variant of a bimanual task in which one arm was moved twice or thrice faster than the other arm. Upon reaching proficiency, participants were asked to switch to the converse pattern in which the latter arm was moved twice or thrice faster than the former one. Results showed that training of one variant of the task led to a negative effect on the performance of the converse variant^9^. This inspired a new model of motor memory, consisting of an abstract effector-independent and an effector-specific layer.

Although numerous studies have explored the behavioral features of motor skill transfer, its underlying neural mechanisms remain largely under-investigated. Functional MRI studies have shown that changes in the activation of the primary somatosensory (S1) and motor cortex (M1) as well as the supplementary motor area (SMA), are linked to transfer of learning, but this effect might be task-specific^12,13^. Interestingly, in unimanual transfer of learning using a sequential motor task, “bilateral” activations in the M1^14^, S1, and dorsal premotor cortex (PMd)^15^ have been observed. The observation of bilateral activation in the sensorimotor areas is an important finding, suggesting that unimanual training may build bilateral representations. However, the activation was found to be stronger in the trained hemisphere as compared to the untrained one^15^. It has been proposed that activation in the untrained M1 may be predominantly mediated by the excitatory input from the trained M1^14^. This may account for positive unimanual transfer. However, when subtasks for each hand have to be conversed in a bimanual condition, this process may induce inter-hand interference requiring inhibitory processes. Thus, both inhibitory and excitatory mechanisms may play a role in transfer of motor learning, depending on the task context.

Apart from the functional brain characteristics, the neurochemical properties of the brain may also play a role in transfer of learning. Investigating the concentrations of neurometabolites has been made possible by the development of advanced magnetic resonance spectroscopy (MRS) techniques. Specifically, the MEGA-PRESS sequence allows quantifying the concentration of γ-aminobutyric acid (GABA) and Glutamate + Glutamine (Glx). GABA is known as the major inhibitory and Glutamate as the major excitatory neurometabolite in the brain. Task-induced modulations of the MRS-assessed GABA and Glx concentrations have been investigated in various motor tasks, including hand grip^16^, sequence learning^17–19^, bimanual coordination^20^, force tracing^21^, and multidigit reaction time^22^. However, to the best of our knowledge, no study has yet investigated the role of inhibitory and excitatory mechanisms in transfer of learning.

Accordingly, on one hand, it has been proposed that maintaining appropriate neural suppression through higher GABA concentrations in perceptual processing regions leads to more distinctive practice-induced perceptual representations^23^. Thus, the transfer of learning might induce an increased level of GABA to increase the distinctiveness between the previously learned and the new to-be-learned tasks. On the other hand, an increased concentration of Glu has been associated with increased cortical excitability, which enhances the learning of new tasks^24^. Therefore, an increased level of Glu might facilitate the acquisition of the new skill and lead to increased transfer of learning ability.

To test these hypotheses, we used a bimanual coordination task to investigate two distinct yet related task variations, requiring individuals to perform rotative movements with both hands simultaneously at prescribed frequencies. One task variation (Task A) required the right hand to move twice or thrice faster than the left hand. The other variation (Task B) required the left hand to rotate twice or thrice faster than the right hand. Using single-voxel magnetic resonance spectroscopy, concentrations of GABA and Glx were determined in two sensory processing regions, i.e., the somatosensory cortex (S1), and a visual motion-sensitive region in the state occipito-temporal cortex (MT/V5). Measures were administered prior to, during, and after the transfer of the motor skill. The selection of S1 as a VOI was motivated by its prominent role in proprioceptive processing^25^. Particularly in the absence of visual feedback, participants are required to rely more on proprioceptive information for task performance and/or learning. Furthermore, previous studies have reported transfer-induced changes in the activation of the S1^15^, making it a potential candidate for neurometabolite investigation in this study. Regarding the MT/V5, its role in tracing a visual target on a PC screen with both hands has been well documented, including fMRI work done by our team^26–29^.

At the behavioral level, we anticipated a duality in skill transfer effect regarding the abstract or general (effector-unspecific) and concrete (effector-specific) levels of the motor representation. On the one hand, we hypothesized a positive skill transfer effect as a result of the high degree of similarity between the two task sets, as indexed by better performance of the un-trained task during transfer as compared to the baseline, i.e., positive effector-unspecific transfer. On the other hand, negative transfer of learning was hypothesized to be dominant because of the reversal of subtask allocation to each hand, i.e., negative effector-specific transfer. This would be indexed as poorer (not even equal) performance when shifting from Task A to Task B as compared to the proficient level obtained in Task A following its practice.

At the neural level, we hypothesized suppression of inter-hand interference via increased GABA concentrations in both S1 and MT/V5 regions upon transition from Task A to Task B. Regarding Glx, we hypothesized that increased Glx concentrations in both S1 and MT/V5 voxels would facilitate learning the new task and improve transfer of learning. This was inspired by previous work showing that increased Glutamate or Glx concentrations are associated with enhanced cortical excitability and facilitation of learning^17,24^. Finally, we expected that individual differences in the concentrations of GABA and Glx would be associated with a successful transfer of the trained skill.

## Materials and methods

### Participants

Fifty-one healthy adults (age range: 18–35 years, mean ± SD: 26 ± 4.07 years) with normal or corrected-to-normal vision were recruited. All the participants were right-handed, according to^30^, and reported no history of psychiatric and/or neuromuscular impairments. Participants were screened for depressive symptoms using Beck’s Depression Inventory^31^ and excluded if they scored higher than 9. Moreover, at the beginning of each session, the Stanford Sleepiness Scale (SSS) was used to subjectively evaluate participants' alertness during the session and exclude participants with insufficient sleep^32^. Participants were randomly assigned to two groups: Experimental group (n = 25) and Control group (n = 26). Two participants were excluded from the study because of technical problems in their MRS acquisition: i.e., poor voxel placement and low signal quality due to lipid contamination, and one participant was excluded because of the inability to perform the task. In the end, the groups consisted of: Experimental group: n = 23, 11 males, age: 25.39 ± 4.41; Control group: n = 25, 11 males, age: 26.67 ± 3.71).

The study was approved by the local Medical Ethics Committee of KU Leuven (study number S58333) in accordance with the Declaration of Helsinki and its amendments. All participants provided written informed consent before the start of the study and received financial compensation for participating.

### Behavioral task and setup

#### Bimanual coordination task (BCT)

**Figure 1:**
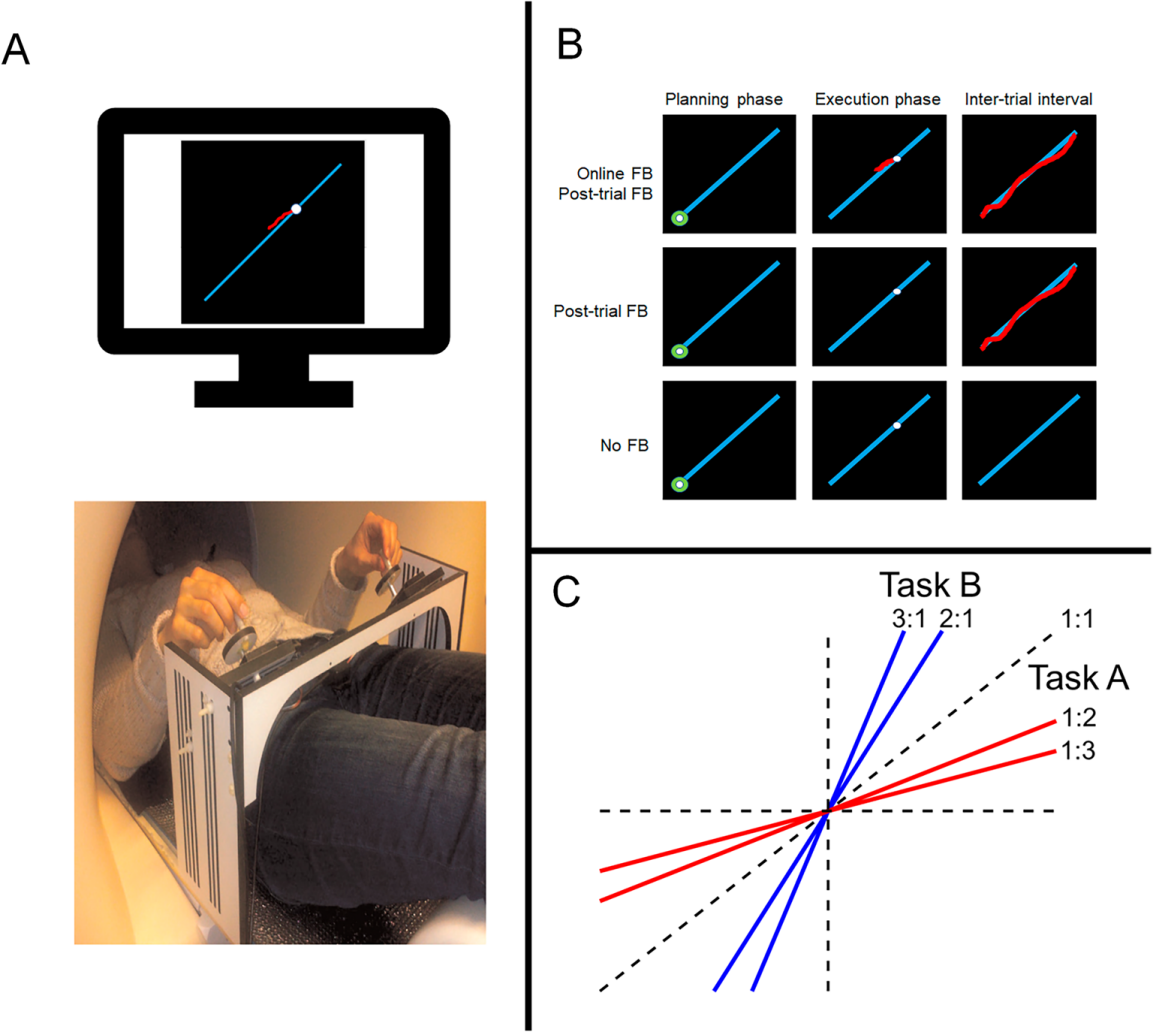
Experimental setup. (**A**) An exemplar participant is in the supine position inside the (real/mock) MRI scanner. (**B**) Participants’ screen view during different phases of task and feedback conditions. In the online FB trials, visual feedback was provided during the trial as well as during the inter-trial interval. In the post-trial FB trials, visual feedback was only provided during the inter-trial interval. In the No FB trials, no feedback was provided during any phases of the trial. (**C**) Bimanual coordination subtasks (Task A: 1:1, 1:2, 1:3, and Task B: 1:1, 2:1, 3:1).

**Figure 2:**
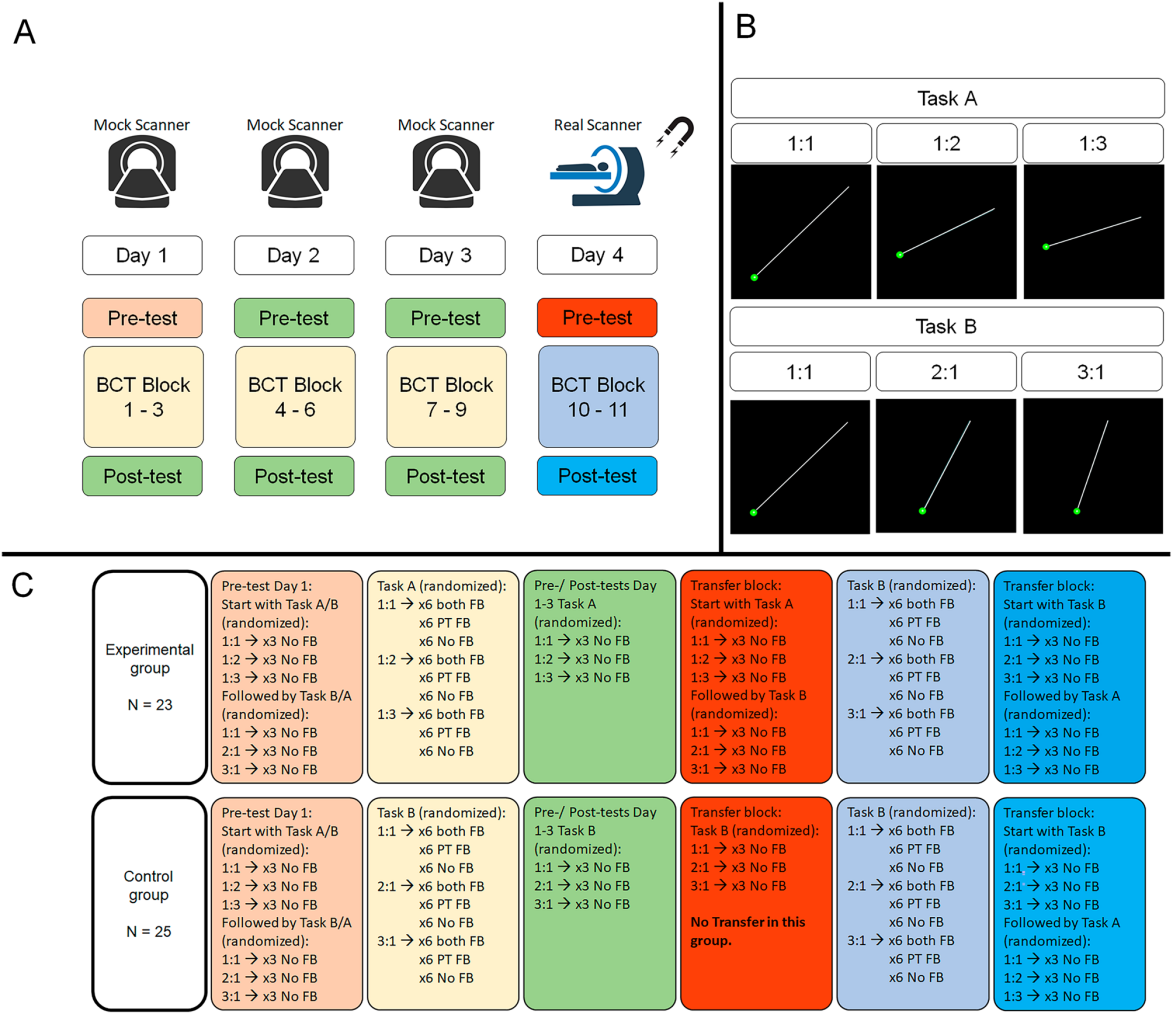
Experimental design. (**A**) The participants went through a four-day training program. The first three days were conducted in the mock scanner and the last day was conducted in the real scanner. Each block of the experiment is color-coded and the content of the block is explained in section C. (**B**) Different hand frequency ratios in Task A and Task B, which all required clockwise rotations. (**C**) Color-coded presentation of the content of each block for the Experimental (N = 23) and Control (N = 25) groups. *BCT* bimanual coordination task, *PT FB* post-trial feedback.

For the behavioral investigation, a computerized visuomotor bimanual tracking task was used^33^. The participants were in a supine position, in either a real or a mock MRI scanner, depending on the session (Fig. 2), facing a computer screen (Fig. 1A). The experimental apparatus consisted of a custom-made, non-ferromagnetic device with two flat discs (diameter of 5 cm) with vertical pegs attached near the edges of the discs. The setup was placed on the participant’s lap and adjusted for their most convenient position. The direction and speed of a cursor shown on the screen were controlled by the rotation of the dials. Turning the right dial clockwise and counter-clockwise moved the cursor to the right and left, respectively, and turning the left dial clockwise and counter-clockwise moved the cursor upwards and downwards, respectively. Hence, the simultaneous clockwise rotation of both dials controlled the movement of the cursor in the top right quadrant (as displayed in Fig. 1C). The objective of the task was to follow a white target dot moving along a blue target line on the screen (Fig. 1B). Angular displacements of the dials were recorded with non-ferromagnetic high-precision optical shaft encoders (HP, 2048 pulses per revolution, 100 samples per sec, accuracy = 0.088°) and were processed online using LabView 8.5 (National Instruments, Austin, Texas, USA). For each trial, the x and y positions of the participant’s cursor were sampled at 100 Hz and recorded for the subsequent offline behavioral analysis conducted in MATLAB R2019b (The MathWorks Inc., Natick, MA).

Each trial consisted of a planning phase, an execution phase, and an inter-trial interval. During the planning phase, the target trajectory (i.e. a blue line) and the target dot (i.e., the white circle) were displayed, and a green lock was placed around the white dot for 2 s. The participants were instructed to remain still and plan their movement during this period. Subsequently, the start of the execution phase was marked by the disappearance of the green lock when the target dot started moving along the target trajectory at a constant speed for a duration of 8 s. During this time, participants were instructed to closely track the target dot by rotating both dials simultaneously. The goal of each trial was to generate the correct direction and speed by turning the dials in order to stay as close as possible to the white target dot. Two types of visual feedback (FB) were provided, namely online FB and post-trial FB. Online FB referred to the provision of information about ongoing performance as indicated via a red cursor, which depicted the current position as well as the positions corresponding to the preceding 1 s during the execution phase. Post-trial FB referred to the information provided after the trial was completed during the inter-trial interval of 2 s and displayed the actual full trajectory of the cursor on top of the ideal trajectory, hence revealing the discrepancy between the produced and the required movement. This justifies our choice to assess neurochemical concentration in regions for movement-related proprioceptive and visual information processing.

The task could be performed according to different frequency ratios, which were visualized by the slope of the target line. A target line with a 45° slope indicated a 1∶1 frequency ratio, whereby both hands were required to rotate at equal speeds. We used the convention of referring to the left hand first and the right hand second, i.e. L:R. For example, a 1∶2 frequency ratio required the right hand to move twice faster than the left hand. Accordingly, two sets of opposite task configurations were defined: Task A, including 1:1, 1:2, and 1:3 frequency ratios, and Task B, including 1:1, 2:1, and 3:1 frequency ratios (Fig. 1C). For all of the ratios in Task A and B, participants needed to rotate the handles in a clockwise manner.

### Experimental design

This study consisted of four sessions, which were spread across a time window of 4.46 ± 0.61 (mean ± SD) days (Fig. 2). Participants in the Experimental group practiced Task A during the first three days and subsequently switched to Task B on Day 4. In contrast, participants in the Control group practiced Task B across all four sessions. Day 1 to Day 3 were carried out in a mock scanner, and Day 4 was performed in a real MR scanner. On Day 1, participants were familiarized with the basic requirements of the task, i.e. information about the dial rotations and the associated cursor movements. No information was provided on how to produce the different frequency ratios. To assess whether participants understood the task, a familiarization block including 10 trials was presented to them. To prevent bimanual learning during the familiarization block, they were instructed not to use both hands simultaneously in the familiarization block.

*Task training (Day 1–3, in the mock MRI scanner)* Each training session started with a short pre-test consisting of 3 trials per frequency ratio presented in random order. During the pre-test, no visual feedback was provided to the participants to avoid any learning effect. For both groups, the pre-test on Day 1 included trials from both Tasks A and Task B. For half of the participants, trials from Task A were presented first, and for the other half, trials from Task B were presented first. On Day 2 and Day 3, the pre-test of the Experimental group included only Task A trials, and the pre-test of the Control group included only Task B trials, corresponding to the to-be-trained tasks.

The pre-test block was followed by three blocks of training. During each block of training, 18 trials per frequency ratio were presented, consisting of 6 No FB trials, 6 trials with only post-trial FB, and 6 trials with both online and post-trial FB (see Fig. 2C). The trials were pseudo-randomized. Each block of training lasted 11 min (54 trials × 12 s ~ 11 min; total duration for 3 blocks ~ 33 min). A 2-min rest interval was provided between blocks to avoid fatigue and discomfort. Following the training, the participants performed a post-test to assess their learning trends. The post-test was the same as the pre-test consisting of 3 trials per the frequency ratio which had been trained during the session, without provision of any type of visual FB.

*Training and Transfer (Day 4, in the real MRI scanner)* On Day 4, first, a pre-test block was administered, with 3 trials per frequency ratio presented in random order. For the Experimental group, the pre-test consisted of trials from both tasks, starting with trials from Task A and then proceeding with trials from Task B. For the Control group, the pre-test only consisted of Task B trials. The pre-test block was followed by two training blocks. During these training blocks, participants in the Experimental group switched to performing the transfer task (i.e., Task B) whereas participants in the Control group continued training on Task B. Subsequently, both groups performed a post-test block consisting of trials from both tasks whereby Task B trials were followed by Task A trials (Fig. 2).

#### Behavioral data analysis

BCT performance was defined as the deviation of each sample point from the nearest target track point. Thus, for each sample (i.e., every 10 ms) of the participant’s trajectory, a point on the target pattern with minimum Euclidean distance from the subject’s trajectory was identified. The final performance score was calculated as the average of the Euclidean distances^34^. Thus, moving away from the target line, or moving too slow or too fast with respect to the target dot would result in a higher average target deviation value, or, in other words, poorer performance.

While the trials in which some type of visual feedback is provided are essential to support the acquisition of the task, they do not clearly depict the actual learning because of the temporary effects of augmented feedback on task performance. Therefore, we chose to only analyze the performance in the No FB trials, which are also consistent with the trials in the pre- and post-tests. The 1:1 ratio was included to help participants appreciate the difference between symmetrical and asymmetrical rotations. However, we were mainly interested in evaluating the acquisition of nonsymmetrical frequency ratios (i.e., 1:2/2:1/1:3), in which the rotation speeds between the two hands were not equal. Therefore, the 1:1 ratio was not included in our analyses. For each subtask, the median of average target deviations of trials in each block was calculated and considered as the measure of participant’s performance in that block.

Importantly, task transfer from task A to B occurred during the pre-test of Day 4 in the Experimental group. Hence, for participants in the Experimental group, the difference between the performance in Tasks B and Task A during the pre-test on Day 4 (Task B − Task A) was defined as the task-transfer performance. Accordingly, higher scores (indicating greater deviation from the target in Task B compared to Task A) referred to the less successful generalization of the previously trained task to the opposite one. This difference measure was separately calculated for 1:2 to 2:1 and 1:3 to 3:1 ratios and was subsequently used in brain-behavior analyses.

### MR acquisition

MR data were acquired at the University Hospital Leuven using a 3 Tesla Philips MRI Achieva dStream scanner equipped with a 32-channel, receive-only head coil. On Day 4, first, a high-resolution, *T*_1_-weighted image was acquired using chemical-shift 3D turbo-field-echo imaging (3DTFE; TE = 4.6 ms, TR = 9.7 ms, 1 × 1 × 1 mm^3^ voxel size, field of view (FOV) = 182 × 288 × 288 mm^3^, 182 sagittal slices, scan duration ≈ 7 min) to capture the anatomical features of the brain.

Subsequently, to obtain baseline concentrations of neurometabolites, MRS scans were collected during the resting state from two voxels of interest (VOIs), namely the S1 and MT/V5. MRS data were acquired using the MEGA-PRESS sequence^35,36^ (TE = 68 ms, TR = 2 s, 2 kHz spectral width). For each MRS scan, 320 averages (160 ON and 160 OFF) were acquired (scan duration = 11 min 12 s). Notably, MRS requires the use of relatively large voxels to ensure an acceptable signal-to-noise ratio (SNR)^37^. Thus, considering the shape and dimensions of each region of interest, the voxel dimensions were set as 25 × 40 × 25 mm^3^ for the left S1 and 40 × 25 × 25 mm^3^ for the left MT/V5 voxel^38^. ON and OFF spectra were acquired in an interleaved fashion, corresponding to an editing pulse at 1.9 or 7.46 ppm, respectively. Prior to each MRS acquisition, an automatic shimming procedure was performed. For both VOIs, 16 unsuppressed water averages were acquired within the same VOI using identical acquisition parameters. Because macromolecules are co-edited (GABA + macromolecules), we refer to the GABA concentration as GABA+. MRS VOIs were identified on a subject-to-subject basis using anatomical landmarks. The S1 VOI was first placed over the hand knob of the left motor cortex and was then moved toward the posterior direction until the postcentral gyrus was covered. Afterward, it was rotated to stay parallel with the cortical surface in the coronal and sagittal plane. For the MT/V5 VOI, we started by screening the slices from lateral to medial on the sagittal view; then, the center of the voxel was placed at the posterior part of the medial temporal gyrus (the conjunction between temporal and occipital cortex). Subsequently, the voxel was rotated to be parallel with the cortical surface in the axial and sagittal planes.

Afterward, a low-resolution short *T*_1_-weighted image was acquired to adjust the voxel locations to compensate for participants’ movements (TE = 2.6 ms, TR = 9.6 ms, 1 × 1 × 1 mm^3^ voxel size, FOV = 182 × 256 × 256 mm^3^, 182 sagittal slices, scan duration ≈ 1.5 min). Participants performed the pre-test block during the acquisition of this short *T*_1_-weighted image.

**Figure 3:**
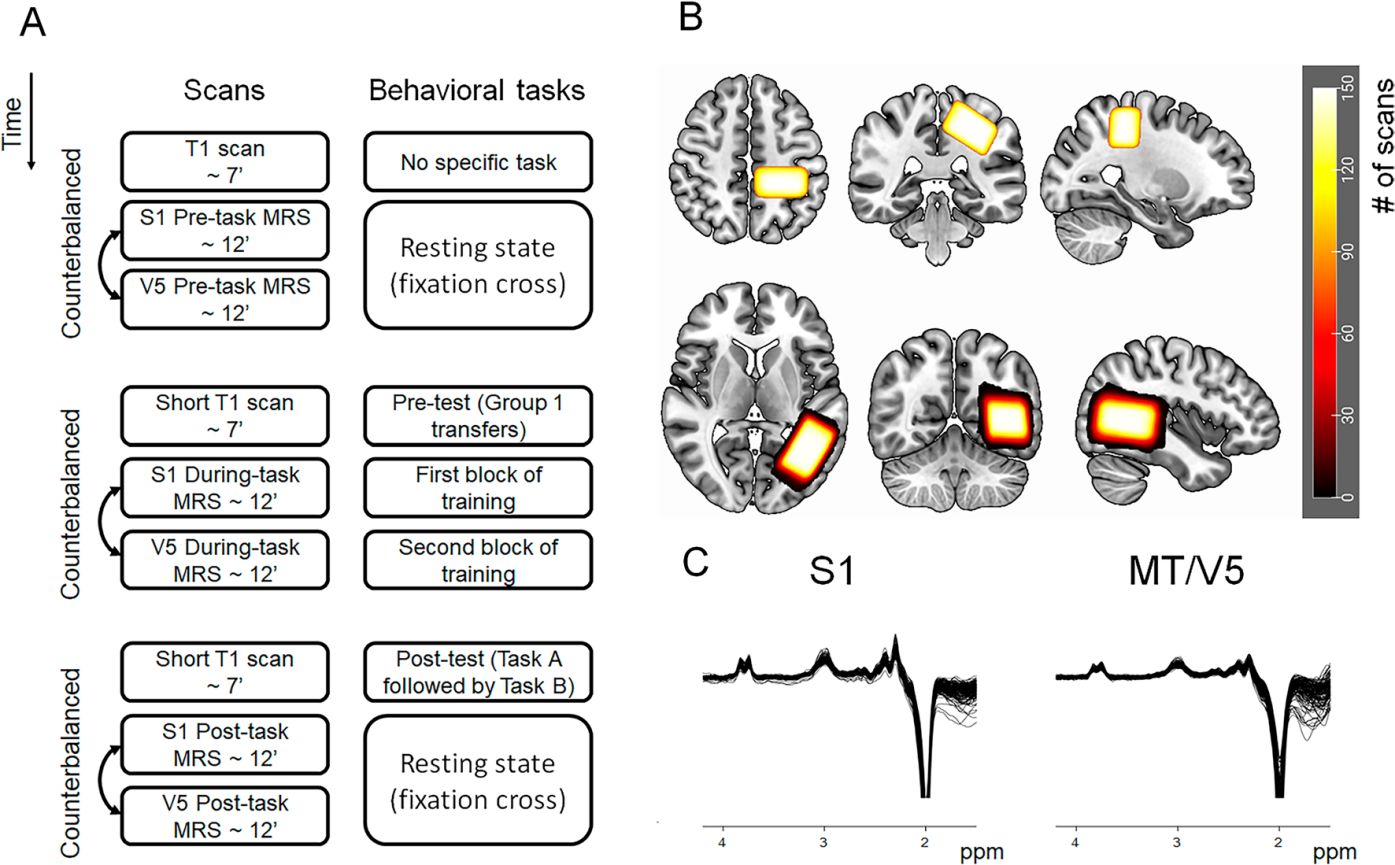
MRI scan procedure. (**A**) Timeline of the MRI data acquisition on Day 4. (**B**) Heatmap of the voxel locations for the S1 and MT/V5 brain regions across all participants in the standard MNI 152 space (radiological view). For each participant, the S1 and MT/V5 VOIs were scanned at three time points (pre-, during-, and post-task), thus, overall each voxel was placed 150 times. (**C**) MRS spectra obtained from the S1 and MT/V5 brain regions.

During each training block, the MRS data were acquired from the S1 or MT/V5 voxels. Subsequently, both groups performed the post-test, where another short *T*_1_-weighted image was collected to adjust the location of the voxels in case of any movement. Finally, resting-state MRS scans were acquired from both VOIs to measure the post-task concentration of neurometabolites. The order of the MRS acquisitions for each VOI was counterbalanced across participants in each group to avoid any effect of acquisition order (Fig. 3A).

#### MRS data analysis

The pipeline of the acquired spectra at all three time points, as well as voxel locations in the MNI space, are presented in Fig. 3. The MRS data were analyzed using the Gannet analysis toolkit (version 3.2.1b)^39^. First, spectral registration was applied for frequency-and-phase correction^40^. Subsequently, the GABA and Glx signals were fitted between 4.2 and 2.8 ppm using a three-Gaussian function, whereas the water signal was fitted using a Gaussian–Lorentzian model. Next, considering that CSF contains a negligible amount of metabolites^41^, and assuming that their concentrations are twice as high in gray as compared to white matter^42^, the concentrations were corrected for tissue fractions within each VOI^43^. To this end, MRS voxels were co-registered to the anatomical images that were used to correctly position the VOIs. If correct VOI positioning was confirmed on a low-resolution short *T*_1_-weighted image acquisition (i.e., for VOIs acquired during and after task performance), the high-resolution *T*_1_-weighted image acquired at the beginning of the session was co-registered to this short anatomical image and was used to ensure proper resolution for the segmentation step. The fraction of gray matter, white matter, and CSF within the VOI was calculated by segmentation of the data using statistical parametric mapping (SPM12) software. In the last step, GABA and Glx concentrations were normalized to the average voxel composition of the whole group^43^. Water was used as a reference compound.

Data were assessed both qualitatively by visual inspection of the spectra for lipid contamination and quantitatively using inspection of values for SNR, frequency drift, and full-width, half-maximum (FWHM) of the modeled *N*-Acetylaspartate (NAA) signal (See Table S3 in the supplementary information). Overall, MRS data from two participants (~ 4% of the acquired data) were excluded because of lipid contamination and low SNR. Thus, our analyses were based on 48 spectra obtained for pre-, during-, and post-task MRS data (Experimental group (n = 23) and Control group (n = 25)).

### Statistical tests

Statistical analyses were carried out using R (version 4.1.2, R Core Team, 2021).

#### Behavioral data

##### Performance on Day 1

To investigate whether there was any difference in the initial performance between the two groups, repeated measures 2 × 2 × 2 (Group [Experimental vs Control] × Ratio [1:2, 2:1 vs 1:3, 3:1] and Task [Task A vs Task B]) ANOVA was conducted in which Group was entered as a between-subjects factor, and Ratio and Task were entered as within-subjects factors.

##### Acquisition from Day 1 to Day 3

To examine the effect of training on the performance in each group, modulations in the average target deviation from Day 1 to Day 3 were assessed. The intention was to investigate whether this reduction in target error was significant and whether task acquisition reached a plateau level. Hence, a 2 × 9 × 2 (Group [Experimental vs Control] × Training block [1 to 9] × Ratio [1:2, 2:1 vs 1:3, 3:1]) ANOVA was conducted to examine the effect of Group as the between-subjects factor and Training block and Ratio as the within-subjects factors. Since the data from some time points were not normally distributed, the nonparametric alternative for ANOVA test from the nparLD package in R^44^ was used.

##### Transfer from Task A to Task B in the Experimental group on Day 4

For the participants in the Experimental group, the first exposure to the un-trained task (i.e., Task B) after the pre-test on Day 1, was during the pre-test on Day 4. Specifically, on this day, the Experimental group was first presented with Task A and then Task B, whereas the Control group only continued performing Task B. First, we investigated whether there was any difference in the performance on the trained task between the two groups. Hence using a 2 × 2 (Group [Experimental vs Control] × Ratio [1:2, 2:1 vs 1:3, 3:1]) repeated measures ANOVA, the performance of participants in the Experimental group on Task A and the performance of participants in the Control group on Task B in both Ratios (i.e., 1:2 and 1:3 (Task A) vs 2:1 and 3:1 (Task B)) were compared. No significant difference between these two measures was expected as the difference in the difficulty level of the tasks was expected to be negligible. Next, we used two tests to investigate the transfer effect: (1) in the Experimental group for which the transfer occurred, a 2 × 2 (Task [Task A vs Task B] × Ratio [1:2, 2:1 vs 1:3, 3:1]) repeated measures ANOVA was performed to compare the average target deviation in Task A and the transfer Task B for both ratios. In this test, both Task and Ratio were entered as within-subjects factors; (2) using a mixed-design 2 × 2 (Group [Experimental vs Control] × Ratio [1:2, 2:1 vs 1:3, 3:1]) ANOVA, the performance of the Experimental group on Task B (2:1 and 3:1) was compared with the performance of the Control group on Task B (2:1 and 3:1) as a reference group. In this test, Group was entered as a between-subjects factor and Ratio was entered as a within-subjects factor. We expected that any significant transfer effects would be reflected as the significant main effect of Group in this test.

##### Transfer from Day 1 to Day 4

To investigate the potential positive effects of training Task A on Task B in the Experimental Group, we examined the participants' performance in Task B (both ratios) on Day 1 and Day 4. During this period, the participants only practiced Task A. Thus, improved performance from Day 1 to Day 4 could be linked to the positive impact of training Task A on performing Task B. To assess this effect, a 2 × 2 (Ratio [2:1 vs. 3:1] × Time [Day 1 vs. Day 4]) repeated measures ANOVA was conducted, with Ratio and Time as the within-subject factors.

#### Neurometabolites modulations

Separate tests were performed to investigate task- or transfer-related modulations in the GABA + and Glx concentrations.

To ensure that any potential task-induced modulations in the neurochemical concentrations were not significantly related to the order of the VOI data acquisition, the effect of scan order (i.e., S1 MRS data acquired before or after MT/V5 MRS data) was investigated using a 2 × 3 (Order [S1 MRS first vs. MT/V5 MRS first] × Time [pre- vs. during- vs. post-task MRS]) repeated measures ANOVA. This analysis was separately performed for the Experimental group and the Control group. As results revealed no significant effect of Time × Order interaction (all *p*s > 0.104), this factor was not included in the further analyses.

To investigate the task-related modulations in the neurochemical concentrations in both groups and VOIs, a 2 × 2 × 3 (Group (Experimental vs. Control) × VOI [S1 and MT/V5] × Time [pre-, during-, and post-task)] mixed-design ANOVA was conducted in which VOI and Time were considered as within-subject factors and Group was considered as a between-subject factor. The Glx data did not follow a normal distribution, hence we used the nonparametric alternative of the ANOVA test with the same design. For both GABA and Glx, the concentrations of neurometabolites were not significantly different between the Experimental and Control group during the resting state before task performance.

#### Associations between task transfer and neurometabolite concentrations

To investigate the associations between behavioral performance and neurometabolite measures, nonparametric partial Spearman correlation analyses were conducted. The behavioral measure of the degree of task transfer was defined as the difference between participants' performance in Tasks A and Task B (Task B − Task A) during the pre-test of Day 4. This measure was calculated for both ratios (1:2 to 2:1 and 1:3 to 3:1) in the Experimental group only.

For all statistical analyses, the level of significance was set at *p* < 0.05, two-sided. To correct for multiple comparisons, Bonferroni’s method was used^45^. *P*-values of ANOVAs were corrected for sphericity using the Greenhouse–Geisser method when Mauchly's test was significant. Partial eta squared (*ƞ*_p_^2^) values were reported to indicate small (≥ 0.01), medium (≥ 0.06), and large (≥ 0.14) effect sizes^46^.

## Results

**Figure 4:**
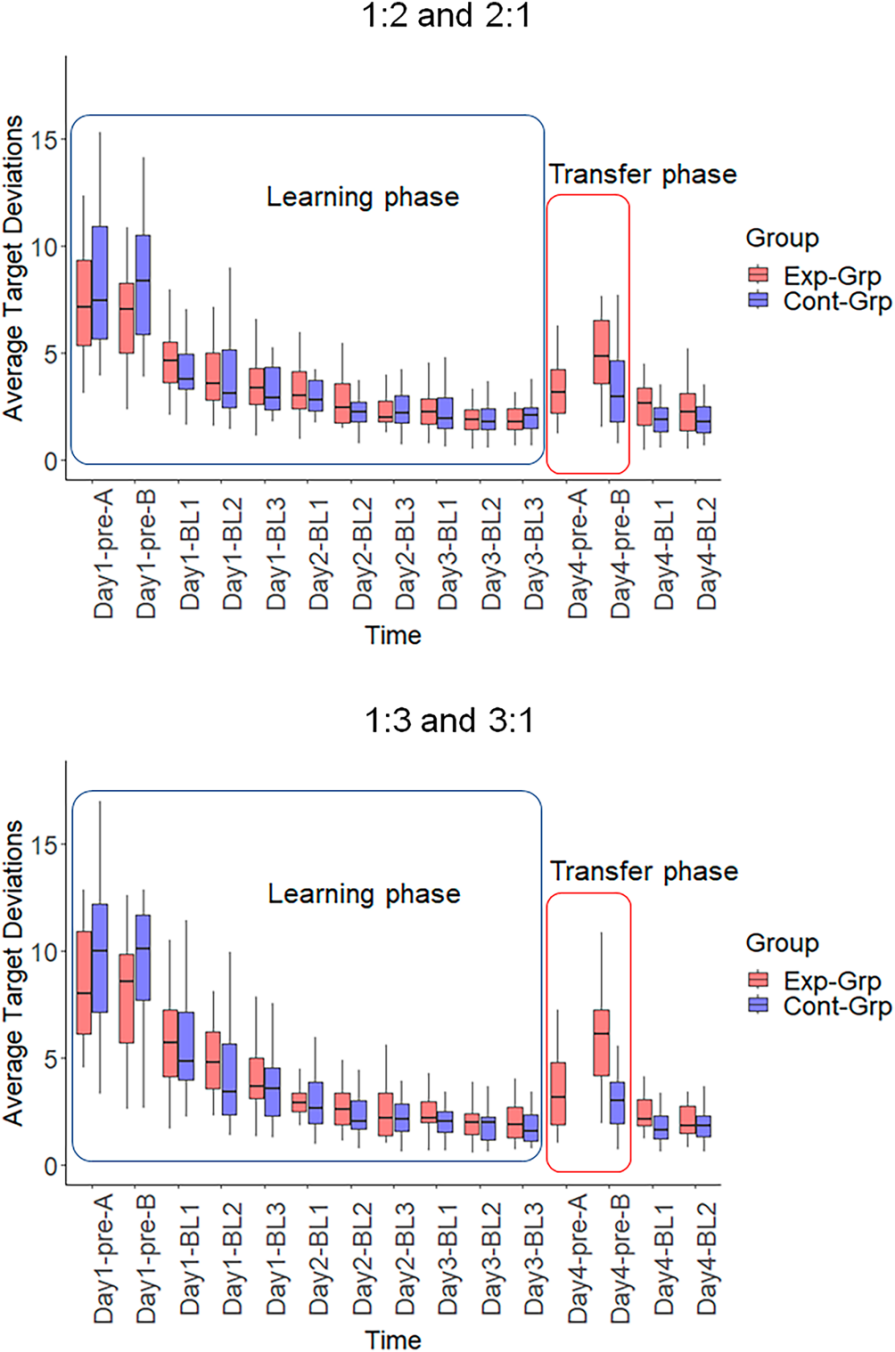
Overview of the participants’ performance in both groups. *Exp-Grp* experimental group, presented with red color, *Cont-Grp* control group, presented with blue color. Day1-pre-A: pre-test of Task A on Day 1, Day1-pre-B: pre-test of Task B on Day 1, Day4-pre-A: pre-test of Task A on Day 4, Day4-pre-B: pre-test of Task B on Day 4, BL1-3: Block1 to Block3. Whiskers indicate the 1.5 × interquartile range of data.

Figure 4 shows the behavioral performance of both groups, during the pre-tests on Day 1 and Day 4 and 11 training blocks from Day 1 to Day 4. The ratios (1:2, 2:1 and 1:3, 3:1) are displayed in separate graphs, with the Experimental group and the Control group coded in red and blue, respectively.

### Pre-test on Day 1

**Figure 5:**
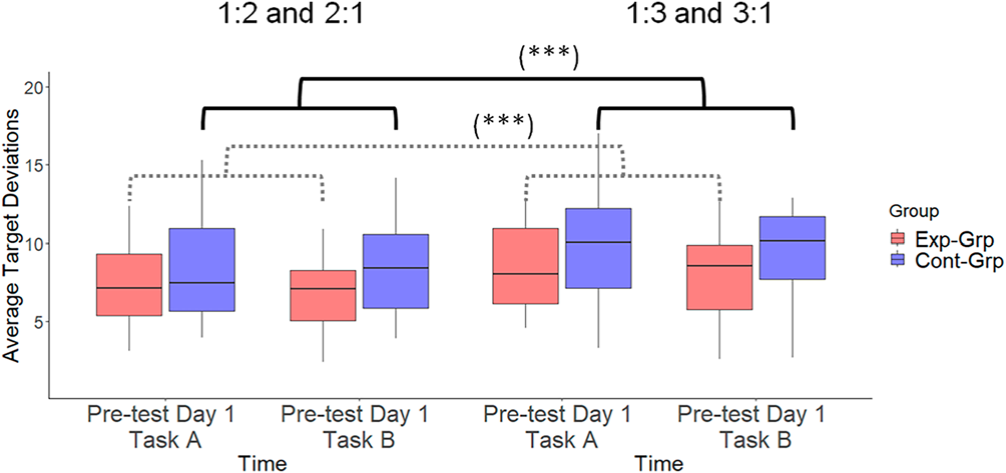
Participants’ performance during the pre-test on Day 1. *Exp-Grp* experimental group, presented with red color, *Cont-Grp* control group, presented with blue color. Whiskers indicate the 1.5 × interquartile range of data. ***p < 0.001.

We compared the performance at baseline (Day 1, Fig. 5) between the two groups by subjecting the data to a 2 × 2 × 2 (Group (Experimental, Control) × Task (Task A, Task B) × Ratio (1:2, 2:1 vs 1:3, 3:1)) mixed-design ANOVA. This analysis revealed neither a significant main effect of Group (*F*(1,46) = 3.262, *p* = 0.077, *ƞ*_p_^2^ = 0.046) nor a significant main effect of Task (*F*(1,46) = 0.481, *p* = 0.492, *ƞ*_p_^2^ = 0.002). However, the main effect of Ratio was significant (*F*(1,46) = 33.490, *p* < 0.001, *ƞ*_p_^2^ = 0.036), indicating that the 1:3 or 3:1 ratios were more difficult than the 1:2 or 2:1 ratios, respectively. Furthermore, no significant interactions between the factors were observed. Hence, no major difference was detectable between the groups’ average levels of performance at the beginning of the experiment.

### Skill acquisition across days of practice

**Figure 6:**
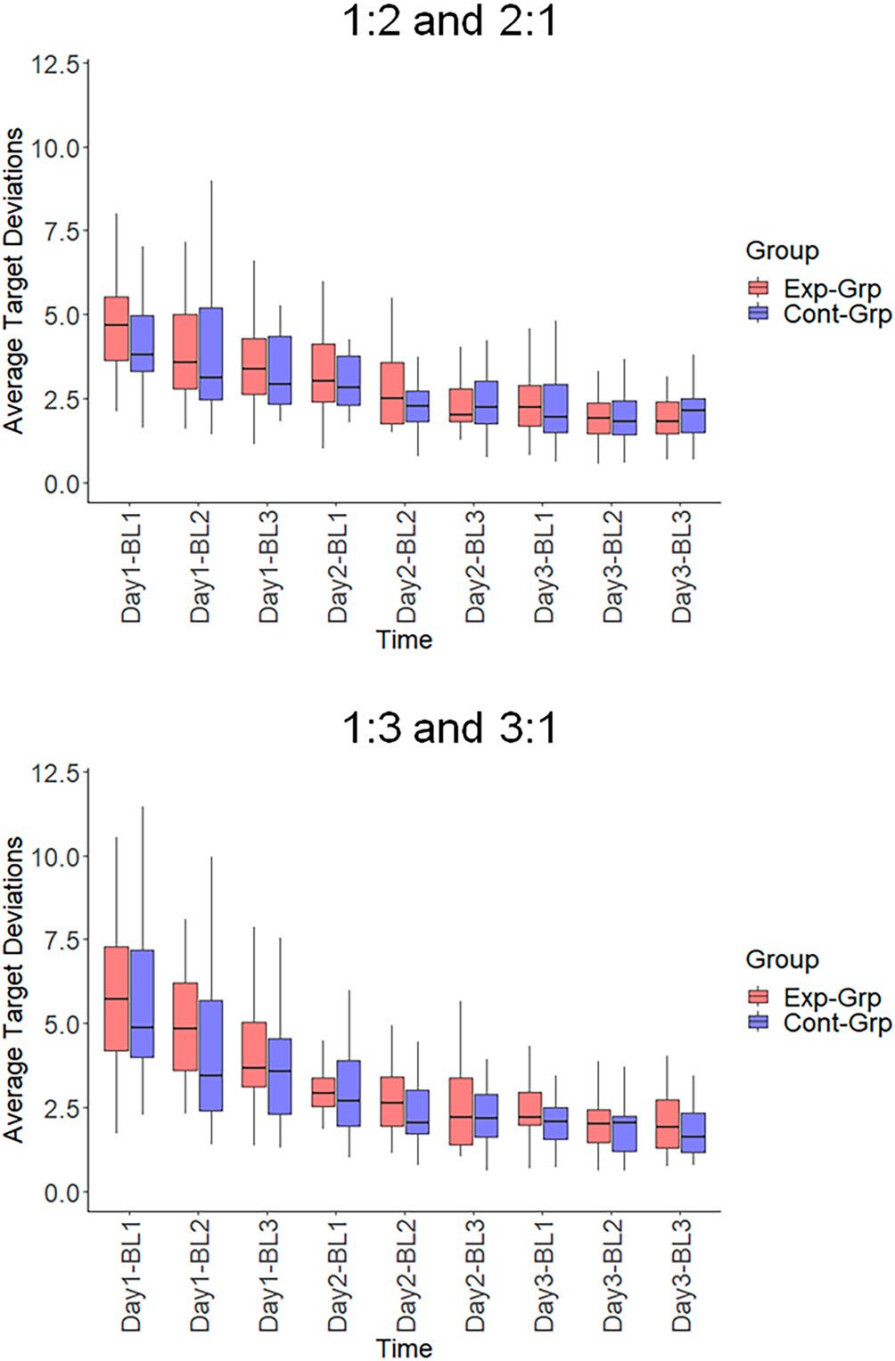
Performance improvement from Day 1 to Day 3 in both groups. Each training day consisted of three training blocks. *Exp-Grp* experimental group, presented with red color, *Cont-Grp* control group, presented with blue color. BL1-3: Block1-3. Whiskers indicate the 1.5 × interquartile range of data.

Training-induced changes in performance were assessed using the nonparametric alternative of a 2 × 9 × 2 (Group (Experimental group performing 1:2 & 1:3; Control group performing 2:1 & 3:1) × Training block (Block 1 to 9) × Ratio (1:2, 2:1 vs 1:3, 3:1)) repeated measures ANOVA. The results showed a significant main effect of the Training block (*F* = 69.662, df1 = 4.320, df2 = Inf, *p* < 0.001), indicating that performance improved over time (see Fig. 6). However, no significant main effects of Ratio (*F* = 0.275, df1 = 1, df2 = Inf, *p* = 0.600) or Group (*F* = 0.974, df1 = 1, df2 = Inf, *p* = 0.324) were observed. The interaction effect of Ratio × Training block was significant (*F* = 2.787, df1 = 6.630, df2 = Inf, *p* = 0.008), indicating that learning rates in 1:3 and 3:1 ratios differed from learning rates in 1:2 and 2:1 ratios. The other interaction effects were not significant.

### Task transfer (pre-test on Day 4)

**Figure 7:**
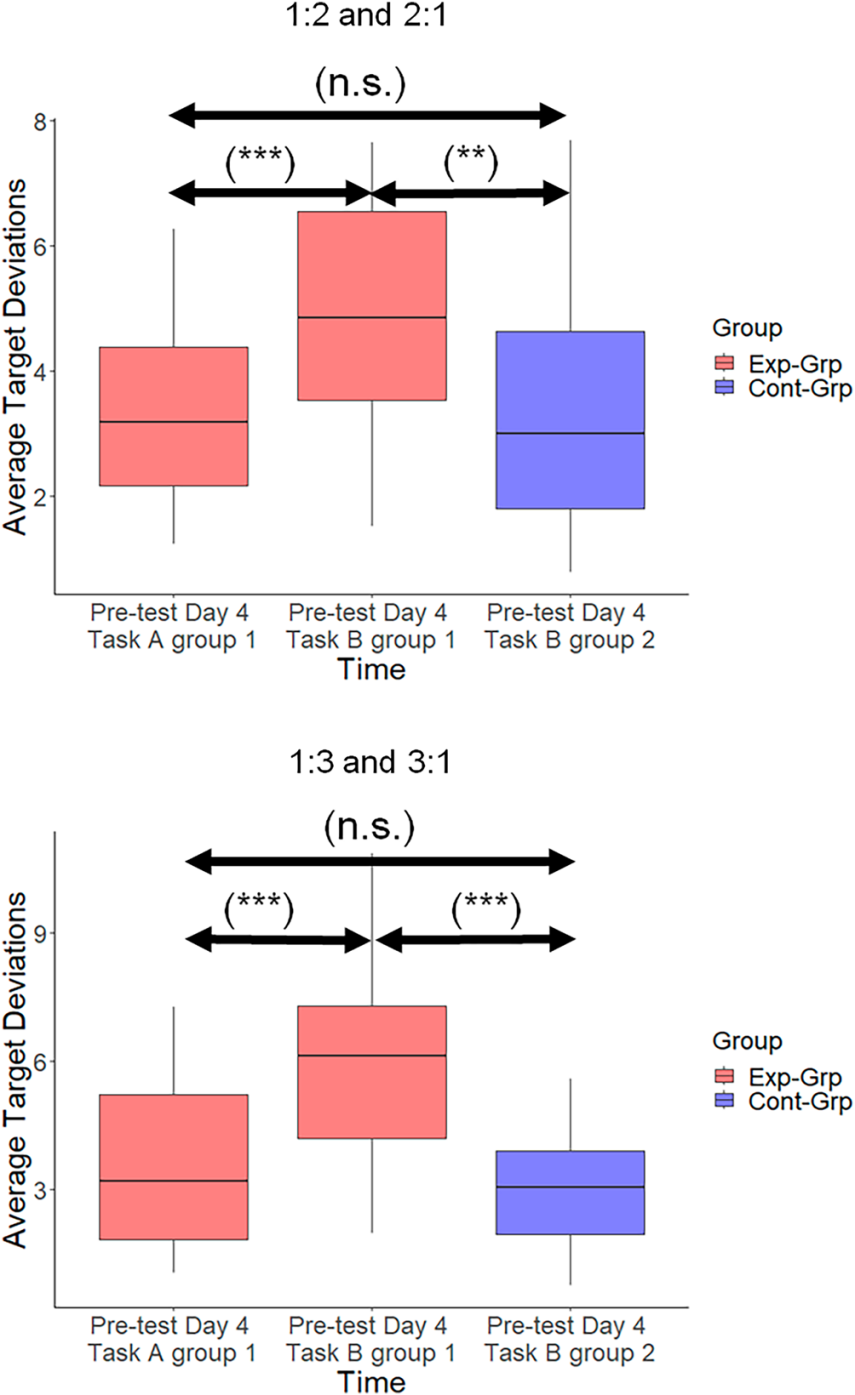
Participants’ performances at the pre-test on Day 4. *Exp-Grp* Experimental group, presented with red color, *Cont-Grp* Control group, presented with blue color. Whiskers indicate the 1.5 × interquartile range of data. ***p < 0.001; **p < 0.01; *n.s.* not significant.

To compare the performance of groups (Task A in Experimental group; Task B in Control group) on their trained task (i.e., prior to task transfer), a mixed-design 2 × 2 (Group [Experimental vs. Control] × Ratio [1:2, 2:1 vs 1:3, 3:1]) ANOVA was conducted on the performance data obtained at Day 4 pre-test (Fig. 7). No significant main effect of Group was observed, indicating that the average target deviation on the trained tasks was not significantly different between Experimental group and Control group (*F*(1,46) = 0.022, *p* = 0.882, *ƞ*_p_^2^ < 0.001). Furthermore, the main effect of Ratio (*F*(1,46) = 0.004, *p* = 0.952, *ƞ*_p_^2^ < 0.001), and the interaction effect of Ratio and Group (*F*(1,46) = 0.668, *p* = 0.418, *ƞ*_p_^2^ = 0.002), were not significant. This suggests that both groups were trained to similar levels on their respective task towards the end of Day 3 and that a priori performance differences between the groups after training, but before transfer, can be excluded.

Subsequently, the degree of transfer was determined via 2 statistical tests. In the first test, the difference between the performance during the trained and untrained (transfer) task in the Experimental group was investigated using a 2 × 2 (Task [Task A vs. Task B] × Ratio [1:2, 2:1 vs. 1:3, 3:1]) repeated measures ANOVA (Fig. 7). We found a significant main effect of Task (*F*(1,22) = 24.947, *p* < 0.001, *ƞ*_p_^2^ = 0.234). This observation revealed higher average target deviations (i.e. poorer performance) in the untrained task (i.e. Task B) compared to the trained task (i.e. Task A). Moreover, we observed a significant main effect of Ratio (*F*(1,22) = 10.016, *p* = 0.004, *ƞ*_p_^2^ = 0.026) as well as a significant interaction effect of Task × Ratio (*F*(1,22) = 7.275, *p* = 0.013, *ƞ*_p_^2^ = 0.015). These findings indicated that performance in the more difficult subtask (i.e., 1:3) was even poorer during the transfer event. In the second test, using a mixed-design 2 × 2 (Group [Experimental, Control] × Ratio [1:2, 2:1 vs 1:3, 3:1]) ANOVA, the performance in Task B was compared between Experimental group and Control group (Fig. 7). A significant main effect of Group was observed (*F*(1,46) = 15.828, *p* < 0.001, *ƞ*_p_^2^ = 0.232). This observation demonstrated that the performance in Task B was poorer in the Experimental group as compared with the Control group. Accordingly, this suggests that transfer in the Experimental group was negatively affected as a result of the alteration in task assignment to each limb. Additionally, the main effect of Ratio (*F*(1,46) = 4.756, *p* = 0.034, *ƞ*_p_^2^ = 0.012), as well as the interaction effect of Ratio × Group (*F*(1,46) = 9.450, *p* = 0.004, *ƞ*_p_^2^ = 0.024) were significant indicating a greater difference in performance between the two ratios (3:1 was more challenging compared to 2:1) in the Experimental as compared to the Control group.

**Figure 8:**
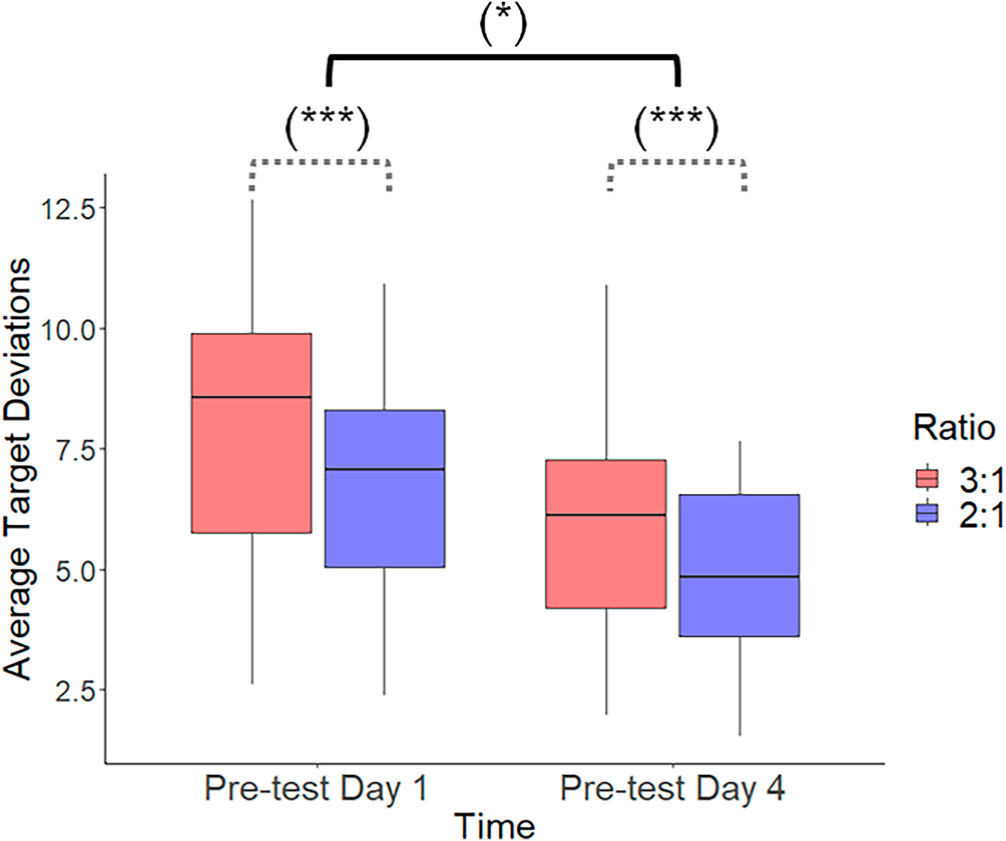
Partial positive transfer of learning in the Experimental group. The performance of the participants in Task B on Day 1 and Day 4 is presented. Between these sessions, they were trained on the opposite task (i.e., Task A). Whiskers indicate the 1.5 × interquartile range of data. ***p < 0.001; *p < 0.05.

Finally, in the Experimental group, we investigated whether performance in Task B was different between the initial pre-test on Day 1 and the pre-test on Day 4 (Fig. 8). The results obtained from the 2 × 2 (Time [Day 1 vs. Day 4] × Ratio (2:1 vs. 3:1) repeated measures ANOVA indicated significant main effects of Time (*F*(1,22) = 406.353, *p* < 0.001, *ƞ*_p_^2^ = 0.883) and Ratio (*F*(1,22) = 7.450, *p* = 0.012, *ƞ*_p_^2^ = 0.146). However, the interaction between Time and Ratio was not significant (*F*(1,22) = 0.095, *p* = 0.761, *ƞ*_p_^2^ < 0.001). These findings suggest that despite the disruption caused by the Task transfer, the training received on Task A had a partially positive impact on the participants' performance in Task B. In other words, participants did not appear to start from scratch when performing Task B on Day 4 and subtask 3:1 was still more challenging to perform than subtask 2:1.

### Task-related modulations in the neurometabolite concentrations

#### *GABA* + 

Results of the 2 × 3 × 2 (Group [Experimental vs. Control] × Time [pre-, during-, post-task] × Voxel [S1 vs. MT/V5]) repeated measures ANOVA revealed a significant main effect of Voxel (*F*(1,46) = 47.929, *p* < 0.001, *ƞ*_p_^2^ = 0.186) indicating a higher GABA + concentration in the S1 as compared to the MT/V5. However, the main effect of Time (*F*(2,92) = 1.480, Greenhouse–Geisser *p* = 0.233, *ƞ*_p_^2^ = 0.006) and interaction effects of Time × Group (*F*(2,92) = 0.947, Greenhouse–Geisser *p* = 0.390, *ƞ*_p_^2^ = 0.004), and Time × Group × Voxel (*F*(2,92) = 2.169, Greenhouse–Geisser *p* = 0.120, *ƞ*_p_^2^ = 0.010) were not significant. For full ANOVA results, see Table S1.

#### Glx

Results of the 2 × 3 × 2 (Group [Experimental vs. Control] × Time [pre-, during-, post-task] × Voxel [S1 vs. MT/V5]) nonparametric ANOVA revealed a significant main effect of Voxel, indicating that Glx concentrations were significantly higher in S1 as compared to MT/V5 (*F* = 27.221, df1 = 1, df2 = Inf, *p* < 0.001). However, the main effect of Time (*F* = 0.022, df1 = 1.862, df2 = Inf, *p* = 0.972) and interaction effects of Time × Group (*F* = 0.857, df1 = 1.862, df2 = Inf, *p* = 0.418), and Time × Group × Voxel (*F* = 0.321, df1 = 1.838, df2 = Inf, *p* = 0.707) were not signifcant. For full ANOVA results, see Table S2.

**Figure 9:**
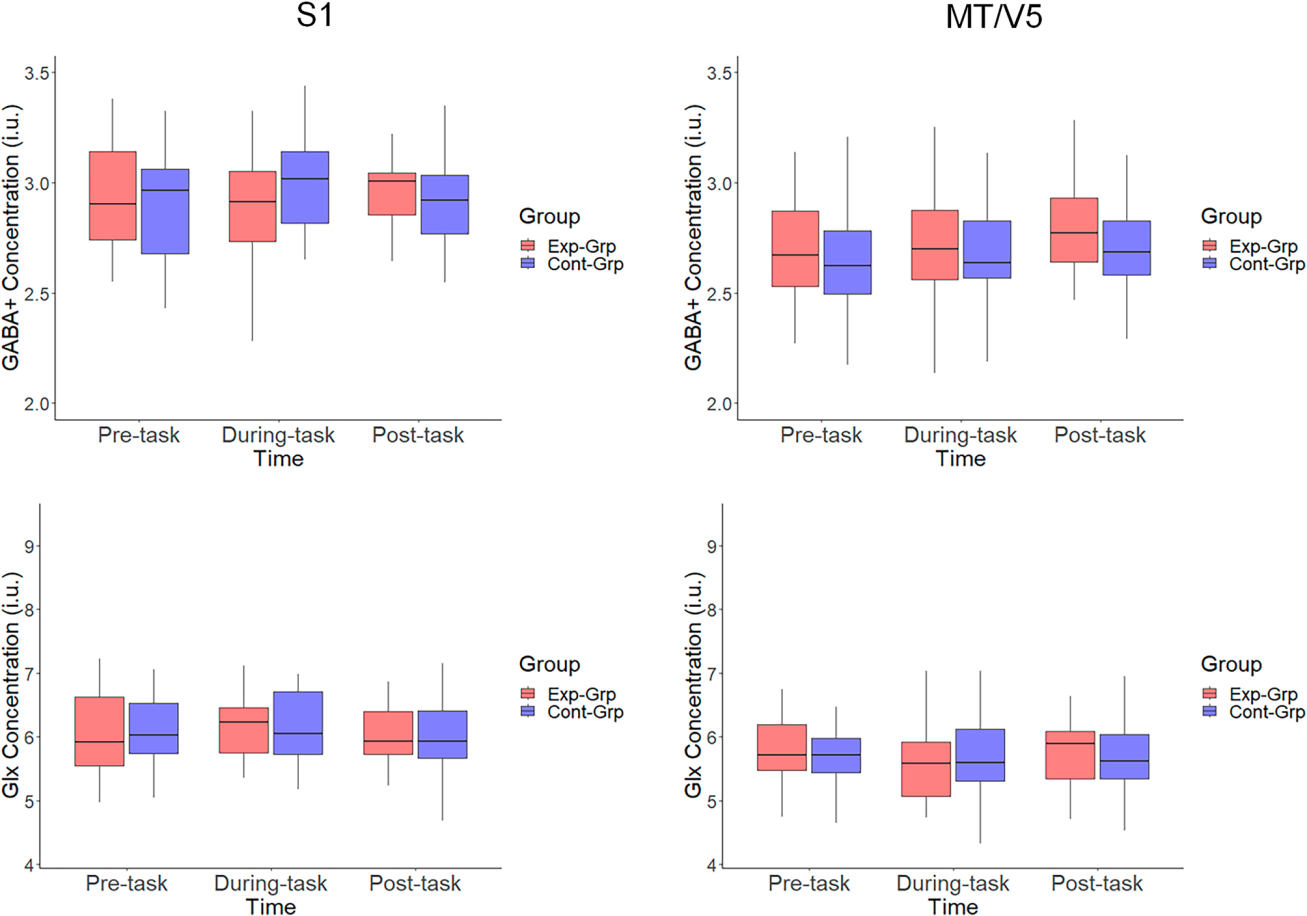
GABA + and Glx concentrations, prior to, during, and after the task performance on Day 4. *Exp-Grp* experimental group, presented with red color, *Cont-Grp* Control group, presented with blue color. Whiskers indicate the 1.5 × interquartile range of data.

In summary, these observations suggest that neurometabolite concentrations were not significantly modulated as a result of task performance as compared to rest (i.e., before and after task performance, see Fig. 9).

### Brain-behavior associations

#### *Associations between GABA* + *concentrations and task transfer*

**Figure 10:**
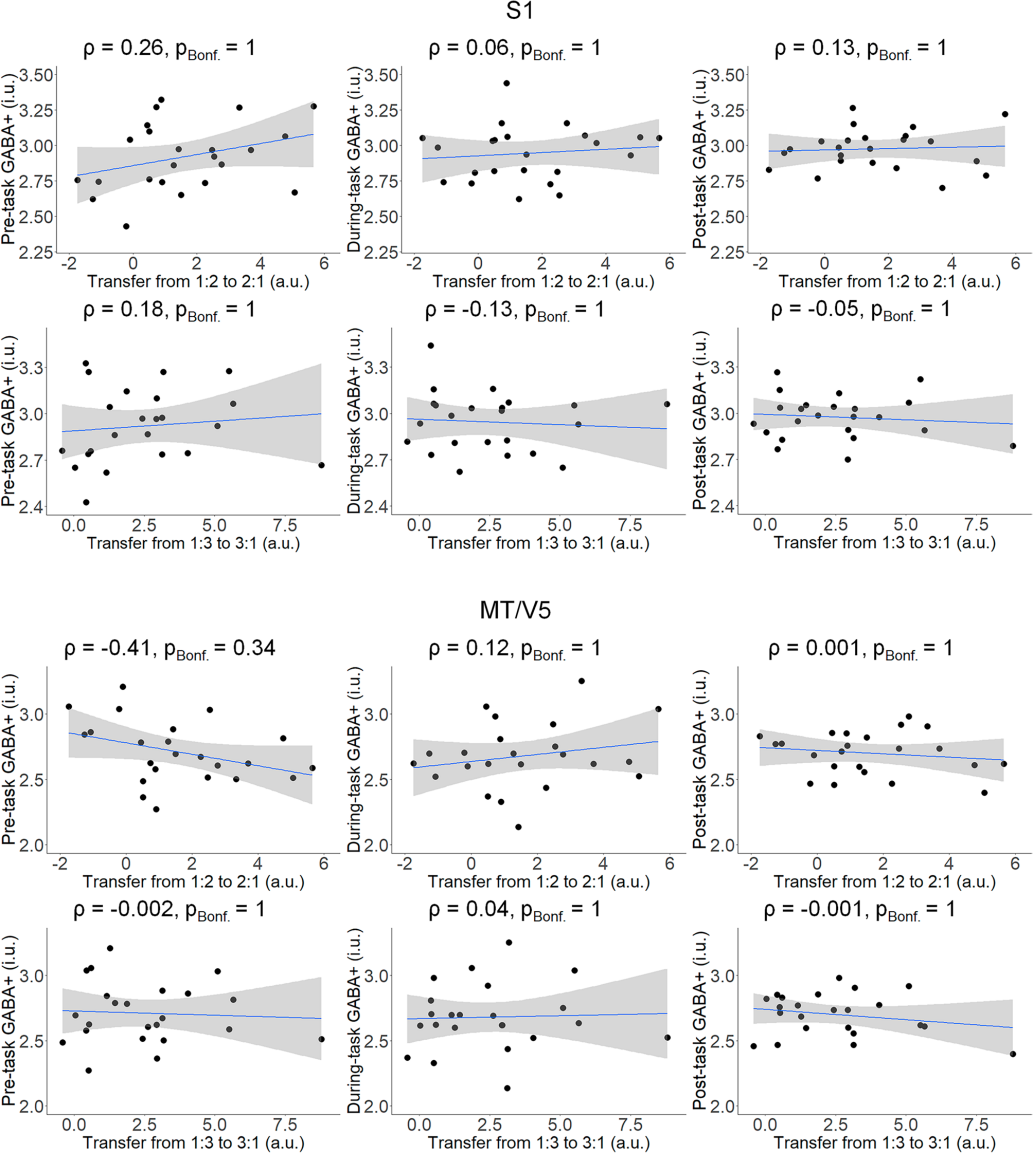
Associations between GABA + concentrations obtained at pre-, during-, and post-task from both voxels of interest (i.e., S1, MT/V5) and task transfer performance in both ratios (1:2 to 2:1, 1:3 to 3:1) in the Experimental group (n = 23). A linear regression line with the 95% CI is added for illustration purposes. *ρ* Spearman’s rho, *p*_*Bonf.*_ *p* after Bonferroni’s correction for 6 comparisons, *i.u* institutional unit; *a.u.* arbitrary unit.

Figure 10 presents the results of the correlation analyses between the GABA + concentrations prior to, during, and after the performance of the task in both voxels and task transfer performance in both ratios. No significant associations were found between the concentrations of GABA + in either of the voxels and task-transfer performance in both ratios.

#### Associations between Glx concentrations and task transfer

**Figure 11:**
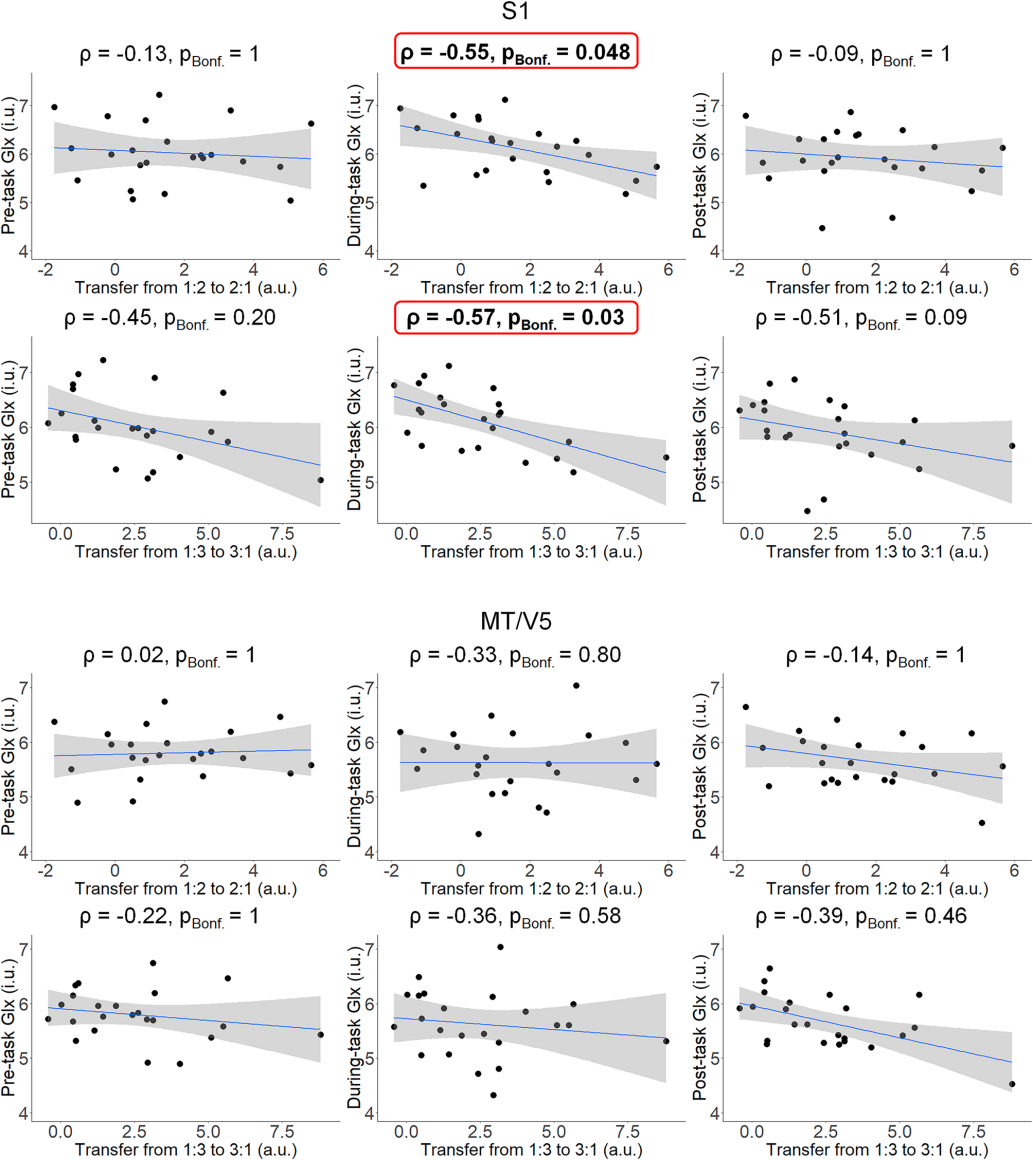
Association between the Glx during pre-, during-, and post-task and task transfer performance in both ratios (1:2 to 2:1, 1:3 to 3:1) and for both voxels (S1, MT/V5) in the Experimental group (n = 23). A linear regression line with the 95% CI is added for illustration purposes. *ρ* Spearman’s rho; *p*_*Bonf.*_ p after Bonferroni’s correction for 6 comparisons (Significant correlation is indicated in bold and red boxes); *i.u.* institutional unit, *a.u.* arbitrary unit.

The Glx concentrations in the S1 voxel during task performance were negatively correlated with task transfer performance in both ratios (1:2 to 2:1 transfer: *ρ* = − 0.55, p_Bonf._ = 0.048; 1:3 to 3:1 transfer: *ρ* = − 0.57, p_Bonf._ = 0.03). This signified that participants with higher Glx concentrations, as obtained during task performance, were more successful with transfer of Task A to Task B. This association was not significant for Glx during the resting state conditions (pre- and post-task). Furthermore, no significant associations were found between the Glx concentrations in the MT/V5 and transfer success (Fig. 11).

#### Difference between the correlation coefficients

Results of Fisher's z-test for comparing correlations using Spearman’s correlation revealed that for the 1:2 to 2:1 task transfer, the correlation between transfer performance and S1 Glx concentration measured during the task was significantly different from the correlations obtained for the post-task and marginally not significant for pre-task Glx concentrations (*z* = 1.67, *p* = 0.047 and *z* = 1.54, *p* = 0.062, respectively). Also, for the 1:2 and 2:1 task transfer, the correlations between task transfer performance and pre- and post-task S1 Glx concentrations were not significantly different (*z* = 0.13, *p* = 0.45). Investigating the same relations for 1:3 and 3:1 task transfer, we found no significant difference between the strength of the correlations between pre-, during-, and post-task Glx and task transfer performance (all *p*s > 0.40). The aforementioned results demonstrated that for 1:2 to 2:1 task transfer, the task transfer performance appeared to exhibit almost a higher correlation with the S1 Glx concentration during task performance than at rest.

## Discussion

We investigated inter-manual skill transfer during bimanual task learning. Specifically, we aimed to determine whether participants could transfer their acquired bimanual coordination skills to perform the task with opposite effector assignments following a training period. This is reminiscent of a transfer from left to right steering wheel driving, or vice versa. Our results demonstrated that participants were unable to fully transfer their acquired skills to the converse coordination pattern, and their performance was inferior to that of the control group, not undergoing task transfer. Furthermore, we explored the neural mechanisms underlying this negative transfer effect by investigating the GABA and Glx concentrations in the S1 and MT/V5 brain regions. At the group level, we observed no significant task- or transfer-related modulations in the concentrations of GABA and Glx. However, at the inter-individual level, we found a positive association between the task-induced concentration of Glx and the ability of participants to transfer their learned skills. This association was evident in the somatosensory cortex (S1), whereby higher concentrations of Glx were linked to better (or less negative) transfer of learning.

### Learning trend from Day 1 to Day 3

Regarding task performance, our results did not show systematic differences in levels of performance between the two groups at the beginning of the experiment. However, the task with the larger ratio (1:3/3:1) was more difficult, as indexed by greater error as compared to the other ratio (1:2/2:1). The task variant with a 1:1 ratio necessitated synchronous bimanual coordination with both hands moving at the same speed. As the ratio increased, the required hand movements became increasingly asymmetric. This required participants to overcome their inherent tendency to execute synchronous movements^33,47–49^. The greater the extent of asymmetry between the hands’ speed, the greater the challenge.

Nonetheless, significant practice effects were detected in both groups. Specifically, the average target deviation consistently decreased over days of practice and reached a plateau level by the end of Day 3. This confirmed that both tasks were well-trained prior to the transfer of learning intervention on Day 4. The extended training most likely maximized the interference effect of the pre-learned task on the performance of the new task variant in the Experimental Group.

### Incomplete transfer of learning

The level of task performance in the Experimental group was disturbed at the pretest on Day 4 when a similar task with reversed subtask allocations (Task B) had to be performed. In the Experimental group, performance of Task B following task transfer was not only inferior to the performance on the trained task (Task A) but was also worse than the performance exhibited by the Control group in Task B. Notably, despite this negative effect, performance on the transferred task was better than the initial performance on that task at the pretest on the first day. On one hand, this observation indicates that interference was manifest at the effector-specific level of the memory representation of movement. In other words, learning a new motor skill requires the establishment of a corresponding motor memory level that represents the specifically employed effectors^9–11^. Since the participants failed to perform the transfer task as accurately as the trained task, we conclude that they failed to realize full transfer of learning. On the other hand, despite the decline in performance, participants still showed better performance as compared to the initial performance of Task B (on Day 1) before starting to learn Task A, indicating a positive transfer effect. This suggests some degree of generalizability in their learning, possibly mediated by the more abstract layer of the motor representation^6–8,50,51^.

Although observing both positive and negative transfer of learning may seem paradoxical at first sight, it is consistent with a dual-layer memory representation of movement. Developing a new motor skill involves the establishment of motor memory at both abstract and effector-specific levels. Converging evidence suggests that the positive impact of training one task on a similar task is related to the encoding of movement specifications at an abstract level^8,52–54^. In principle, the overall movement objectives, such as the relative speed of the hand movements, are possibly incorporated in the abstract layer of motor memory. This interpretation aligns with the principles of the identical elements theory, which postulates that the closer the task components are to each other, the greater the expected transfer effect^55^. Additionally, common elements require shared features of information processing for task execution, thereby promoting positive transfer^5^.

Whereas the abstract layer of motor memory explains the positive transfer effect, it fails to account for the observed negative transfer. Vangheluwe, Suy^9^ and coworkers proposed the integration of a complementary effector-specific layer into the motor memory model. Accordingly, it has been suggested that the effector-specific layer is reflective of joint- and muscle-related movement information^9,56^. When switching to a task with a converse allocation of the subtasks to each hand, previously developed effector-specific memory representations may cause interference and potentially lead to negative transfer and poorer performance^9^. Previous work has shown that such negative transfer is equally evident when shifting from a right hand faster to left hand faster arrangement as compared to a shift from left hand faster to right hand faster arrangement^9^.

The present findings are consistent with the challenges associated with switching between right-hand and left-hand car driving and have implications for human factors approaches. Such a switch requires an adjustment of the driving habits and it takes some time to become familiar with the new layout of the car, including the traffic flow on the road as well as the altered placement of some controls, such as the gear shift, the control of the turn signals and windshield wipers. To reduce the performance errors associated with negative transfer effects, the car driver is advised to temporarily shift from an automatic to a controlled processing strategy in order to re-integrate these altered task assignments for each hand into the novel driving task. With patience and practice the negative transfer effects can be overcome and will no longer be life threatening.

### Task- or transfer-related modulation in the neurometabolites

In the Control group, the participants continued training on Task B during Day 4 as they did during the first 3 days. Therefore, the observed changes in the neurometabolite concentrations from the resting state to the task condition reflect correlates of skilled task performance rather than (transfer of) learning. Our results showed no significant changes in the concentrations of GABA and Glx in both S1 and MT/V5 voxels between task performance and rest conditions. The existing literature on task-induced changes in the GABA/Glx concentrations is relatively limited and lacks consistency. In the sensory domain, several studies have explored GABA modulations in response to repetitive tactile stimulation. For instance, while Heba, Puts^57^ did not find an effect of sensory stimulation on the GABA concentrations in the primary sensorimotor cortex (SM1), Lea-Carnall, Williams^58^ reported decreases in the SM1 GABA concentration. Additionally, studies investigating GABA modulation in response to motor task performance and motor learning have also yielded mixed findings. While decreases in the SM1 GABA concentrations have been reported during hand clenching^59^, execution of a multi-limb reaction time task^22^, uni-manual force tracing task^21^ and uni-manual sequence learning^19^, reports of lack of GABA modulations during motor sequence learning also exists^17,60^.

The inconsistent evidence may arise from differences in the task paradigms and study designs across studies. The lack of significant GABA and Glx modulations in the present study might be attributed to a saturation of learning towards Day 4 when participants in the Control group performed the trained task with high proficiency. Therefore, no additional learning occurred, which might explain why we did not detect any dynamic neurometabolic changes during Day 4.

In the Experimental group, participants were to perform the opposite task variant on Day 4 (Task B). The results showed no significant modulations of GABA/Glx in either the S1 or MT/V5 regions during this transfer event at group level. This is inconsistent with our apriori hypothesis expecting that the development of an effector-specific memory during training on Task A would cause interference in performing the conversed task allocation required for Task B, which in turn would result in an upregulation of GABAergic signaling to suppress the pre-established memory and promote the formation of the new motor memory. Several reasons may explain why our a priori hypothesis could not be confirmed. First, dynamic MRS designs suffer from low temporal resolution. To ensure an acceptable signal-to-noise ratio (SNR), a scan sequence of 8 to 12 min is typically required^61^, while the behavioral phenomenon of transfer may have occurred at a faster time scale. The lengthy scan period might have led to habituation to the new task, thereby failing to capture the shorter-term dynamic changes in neurometabolites in response to initial task transfer. In addition to the low temporal resolution, the SNR of the signal in the MEGA-PRESS sequence is proportional to magnetic field strength, volume of the voxel, and the number of signal averages^61^. Hence, to ensure an adequate SNR, we used a relatively large voxel size (i.e., 25 × 40 × 25 mm for the S1 and 40 × 25 × 25 mm for the MT/V5). However, larger voxel sizes may introduce partial volume effects, resulting in the partial inclusion of non-targeted regions. This may have masked the detection of subtle modulations in the (sub)regions of interest. To overcome the aforementioned limitations, access to higher-field MRI scanners is instrumental to boosting spatial and temporal resolutions^17^. It is also possible that the negative transfer observed in Task B performance on Day 4 did not have a detectable impact on the neurometabolite concentration^17^. Finally, we chose to look at two sensory processing voxels and may have missed neurochemical modulation in other voxels, such as in the primary motor cortex.

### Brain-behavior associations

Our findings did not reveal any significant associations between resting-state or during-task GABA concentrations in either the S1 or MT/V5 regions and degree of transfer. However, we found that participants who exhibited higher during-task S1 Glx concentration showed a greater ability to transfer their skill from the trained task to the untrained task (i.e., reduced negative transfer). This relationship was only observed for the S1 region and not for the MT/V5. Higher Glx concentrations have been associated with increased cortical excitability^17,24^, which is known to facilitate motor learning^17^. These may be some of the reasons for why participants with higher Glx concentrations were better in overcoming negative transfer effects. This tentative account requires further interventional follow-up research in which Glx concentrations are experimentally manipulated to investigate their causal effect on task transfer.

### Methodological considerations

In this study, our primary focus was on exploring the dynamic changes in neurometabolites in response to transfer of learning. However, for a more comprehensive understanding of the brain's neurometabolic behavior during learning and transfer, it would have been helpful to also acquire MRS scans prior to the commencement of training on Day 1. Such an approach can provide a broader perspective on neurometabolic modulations in response to learning.

Regarding the MRS data, as explained in the Methods section, the isolated measurement of GABA level using MRS is not feasible and, typically, macromolecules are co-edited alongside with it. Consequently, this co-editing process may impact the sensitivity of MRS measurements to subtle GABA modulations, as well as associations between GABA levels and task transfer capabilities.

## Conclusions

Our findings are consistent with the notion that learning new skills is associated with the development of a two-layered motor memory representation consisting of an abstract (effector-unspecific) level as well as an effector-specific level. The effector-specific component of the motor memory representation might induce a substantial negative transfer of learning, evidenced by a decreased performance when transitioning from the trained to the untrained task variant. These negative interference effects have important human factors implications. Finally, we identified inter-individual associations between the task-induced S1 Glx concentrations and the degree of transfer, which may stimulate future research into Glx as a potential facilitating agent for the transfer of learning. These results shed new light on the complex mechanisms underlying the transfer of learning in relation to neurometabolites.

### Supplementary Information

Supplementary Information.

## Data Availability

The datasets generated during and/or analyzed during the current study are not publicly available due to not having approval from the ethical committee. However, It can be shared by the corresponding author upon reasonable request.
