# Supplementary material for: Neural correlates of transfer of learning in motor coordination tasks: role of inhibitory and excitatory neurometabolites
Source: Sci Rep. 2024 Feb 8;14:3251. doi: 10.1038/s41598-024-53901-8 (PMC10853253; doi:10.1038/s41598-024-53901-8)
Supplement: Supplementary file 1 — Supplementary Information. [file 41598_2024_53901_MOESM1_ESM.docx]

# Supplementary

**Table S1** Results of the GABA data with three-way mixed ANOVA

| Factor | d*f_n_* | d*f_d_* | *F* | *p* | *ƞ*_p_^2^ |
| --- | --- | --- | --- | --- | --- |
| Group | 1 | 46 | .16 | .69 | .001 |
| Voxel | 1 | 46 | 47.93 | <.001 ^a^ | .19 |
| Time | 2 | 92 | 1.48 | .23 | .006 |
| Group × Voxel | 1 | 46 | .61 | .44 | .003 |
| Time × Voxel | 2 | 92 | .86 | .43 | .004 |
| Group × Time | 2 | 92 | .95 | .39 | .004 |
| Group × Voxel × Time | 2 | 92 | 2.17 | .12 | .01 |
| ***Sphericity Corrections*** |  |  | *GGe* | *p[GG]* |  |
| Time |  |  | .98 | .23 |  |
| Time × Voxel |  |  | 1 | .43 |  |
| Group × Time |  |  | .98 | .39 |  |
| Group × Voxel × Time |  |  | 1 | .12 |  |
|  |  |  |  |  |  |

^a^ p-value ≤ 0.05. Abbreviations: df_n_: degree of freedom numerator, df_d_: degree of freedom denumerator, GGe: Greenhouse-Geisser epsilon, p[GG]: p-value adjusted using the Greenhouse-Geisser correction.

**Table S2** Results of the Glx data with non-parametric alternative for three-way mixed ANOVA

| Factor | Statistic | d*f* | *p* |
| --- | --- | --- | --- |
| Group | .11 | 1 | .74 |
| Voxel | 27.22 | 1 | <.001 ^a^ |
| Time | .02 | 1.86 | .97 |
| Group × Voxel | .27 | 1 | .60 |
| Time × Voxel | 3.32 | 1.84 | .04 ^a^ |
| Group × Time | .86 | 1.86 | .42 |
| Group × Voxel × Time | .32 | 1.84 | .71 |
|  |  |  |  |

*a p-value ≤ 0.05. Abbreviations: df: degree of freedom.*

**Table S3** Quality measures of the MRS data

| Voxel | FWHM (mean ± SD) | | SNR (mean ± SD) | |
| --- | --- | --- | --- | --- |
|  | GABA | Glx | GABA | Glx |
| S1 | 18.04 ± .92 | 13.69 ± .91 | 21.76 ± 4.20 | 18.08 ± 3.66 |
| MT/V5 | 19.20 ± 1.18 | 15.52 ± 2.55 | 15.45 ± 2.86 | 12.70 ± 3.33 |

*The results are obtained from 144 scans from S1 and MT/V5. The data includes 23 participants in the Exp. Group and 25 participants in the Cont. Group, scanned pre- , during-, post-task performance.*
